# Blockage of Autophagy Increases Timosaponin AIII-Induced Apoptosis of Glioma Cells In Vitro and In Vivo

**DOI:** 10.3390/cells12010168

**Published:** 2022-12-30

**Authors:** Chu-Che Lee, Jen-Pi Tsai, Hsiang-Lin Lee, Yung-Jen Chen, Yong-Syuan Chen, Yi-Hsien Hsieh, Jin-Cherng Chen

**Affiliations:** 1 Department of Medicine Research, Buddhist Dalin Tzu Chi Hospital, Chiayi 62247, Taiwan; 2 School of Medicine, Tzu Chi University, Hualien 97071, Taiwan; 3 Division of Nephrology, Department of Internal Medicine, Dalin Tzu Chi Hospital, Buddhist Tzu Chi Medical Foundation, Chiayi 62247, Taiwan; 4 Department of Surgery, Chung Shan Medical University Hospital, Taichung 40201, Taiwan; 5 School of Medicine, Chung Shan Medical University, Taichung 40201, Taiwan; 6 Institute of Medicine, Chung Shan Medical University, Taichung 40201, Taiwan; 7 Clinical Laboratory, Chung Shan Medical University Hospital, Taichung 40201, Taiwan; 8 Department of Medical Research, Chung Shan Medical University Hospital, Taichung 40201, Taiwan; 9 Department of Neurosurgery, Dalin Tzu Chi Hospital, Buddhist Tzu Chi Medical Foundation, Chiayi 62247, Taiwan

**Keywords:** glioma, timosaponin AIII, apoptosis, autophagy, LC3-II

## Abstract

Timosaponin AIII (TSAIII), a saponin isolated from Anemarrhena asphodeloides and used in traditional Chinese medicine, exerts antitumor, anti-inflammatory, anti-angiogenesis, and pro-apoptotic activity on a variety of tumor cells. This study investigated the antitumor effects of TSAIII and the underlying mechanisms in human glioma cells in vitro and in vivo. TSAIII significantly inhibited glioma cell viability in a dose- and time-dependent manner but did not affect the growth of normal astrocytes. We also observed that in both glioma cell lines, TSAIII induces cell death and mitochondrial dysfunction, consistent with observed increases in the protein expression of cleaved-caspase-3, cleaved-caspase-9, cleaved-PARP, cytochrome c, and Mcl-1. TSAIII also activated autophagy, as indicated by increased accumulation of the autophagosome markers p62 and LC3-II and the autolysosome marker LAMP1. LC3 silencing, as well as TSAIII combined with the autophagy inhibitor 3-methyladenine (3MA), increased apoptosis in GBM8401 cells. TSAIII inhibited tumor growth in xenografts and in an orthotopic GBM8401 mice model in vivo. These results demonstrate that TSAIII exhibits antitumor effects and may hold potential as a therapy for glioma.

## 1. Introduction

Glioma is a common and aggressive form of brain cancer, accounting for 15% of all brain cancers [1]. The fifth edition of the WHO classification of tumors of the Central Nervous System (WHO CNS5) classified glioma into astrocytoma (IDH-mutant), oligodendroglioma (IDH-mutant), and glioblastoma (IDH wild type, grade IV) [2] The current standard treatment for all glioma is surgical resection in combination with radiation therapy and chemotherapy [3]. Surgical removal of the tumor results in higher survival. High-dose steroids can be used to decrease symptoms and reduce swelling [4]. Without treatment, survival following glioblastoma diagnosis is only three months. With treatment, survival is 14–15 months, with only 10% of patients surviving longer than five years [5]. 

Autophagy is the self-devouring of cells to remove unnecessary or dysfunctional components, such as damaged organelles and long-lived or malformed proteins, and helps to maintain cell homeostasis [6]. This autophagy process requires some important proteins, including microtubule-associated protein light chain 3 (LC3), Beclin-1 p62 (SQSTM1) and Lysosome-associated membrane protein 1 (LAMP1), and it is involved in autophagosome formation and degradation [7,8,9]. Induction of autophagy by various stressors promotes the conversion of LC3-I to LC3-II, which then becomes linked to p62, integrated into the autophagosome, and finally degraded in the autophagolysosome [10]. There is evidence that autophagy activation or inactivation is implicated in tumorigenesis in a variety of cancer types, such as esophageal squamous carcinoma [11], ovarian cancer [12] and neuroblastoma cells [13]. Intrinsic apoptosis is a programmed and regulated form of cell apoptosis executed by induction of the apoptosome complex containing cytochrome c, APAF1, and caspase-9. After activation, caspase-9 activates caspase-3 and PARP expression [14]. Mcl-1 belongs to the anti-apoptotic Bcl-2 family of proteins and effectively inhibits tBid-induced cytochrome c release from the mitochondria to the cytosol [15]. Emerging evidence demonstrates interactions among the crucial proteins of autophagy and apoptosis, which helps elucidate the molecular mechanisms of the crosstalk between apoptosis and autophagy. This includes p62/caspase-8 [16], Bcl-2/Beclin-1 [17] and Atg12/Mcl-1 [18]. Therefore, autophagy plays an inducing apoptosis or by directly suppressing apoptosis in tumor progression [19]. 

Timosaponin AIII (TSAIII) is a steroidal saponin found in the rhizomes of *Anemarrhena asphadeloides* [20]. Of the many steroidal saponins found in this medicinal plant, the biological activity of TSAIII is among the highest [21]. TSAIII has been used to inhibit inflammation [21], apoptosis [22], autophagy [23], and metastasis in human tumor cells [24]. TSAIII induces cell cycle arrest at G2/M and promotes DNA damage through activation of the p38MAPK pathway, thereby contributing to apoptosis in breast cancer cells [25]. TSAIII exerts anticancer effects by inducing activation of c-Jun N-terminal protein kinase (JNK) and extracellular signal related kinase (ERK) in human melanoma A375-S2 cells [26]. TSAIII also was observed to promote autophagy by targeting LC3-II/p62 and inhibiting the PI3K/mTOR pathway in Jurkat cells [23]. Whether TSAIII exerts such antitumor effects in glioma cells remains to be investigated. This study aims to determine whether TSAIII affects apoptosis, mitochondrial function, and/or autophagy in glioma cells.

## 2. Materials and Methods

### 2.1. Chemicals and Reagents

Timosaponin AIII (purity ≥ 98.0%) was purchased from ChemFaces (Wuhan, Hubei, PRC). DMEM/RPMI-1640 medium for cell culture, fetal bovine serum (FBS), and penicillin/streptomycin were purchased from Thermo Fisher Scientific, Inc (Waltham, MA, USA). (3-(4,5-Dimethylthiazol-2-yl)-2,5-diphenyltetrazolium bromide (MTT) and acridine orange were purchased from Sigma-Aldrich (Louis, MO, USA). Annexin V, Dead Cell Assay kit, and MitoPotential kit were purchased from EMD Millipore Corporation (Billerica, MA, USA). Primary antibodies against cleaved-caspase 3 (9668S), cleaved-caspase-9 (9508S), and cleaved-PARP (9542S) were purchased from Cell Signaling Technology Inc. (Danvers, MA, USA). Antibodies against cytochrome c (sc-13156), Mcl-1 (sc-12756), LAMP-1 (sc-20011), and β-actin (sc-47778) were purchased from Santa Cruz Biotechnology (Dallas, TX, USA). The p62 (NBP1-48320) and LC3-II (NB100-2220) antibodies were purchased from Novus Biologicals (Centennial, CO, USA). Antibody-Ki-67 (ab15580) for IHC staining was purchased from Abcam plc (Cambridge, U.K.).

### 2.2. Cell Lines and Culture Conditions 

Human glioblastoma cell lines GBM8401 (BCRC Number: 60163) and M059K (BCRC Number: 60381) and normal astrocyte cell line CTX TNA2 (BCRC Number: 60547) were purchased from Bioresources Collection and Research Center, Food Industry Research and Development Institute (Hsinchu, Taiwan). GBM8401 cells were maintained in RPMI 1640 medium. M059K cells were maintained in Dulbecco’s Modified Eagle’s Medium and Ham’s F12 medium (DMEM/F12) with 2.5 mM L-glutamine adjusted to contain 1.5 g/L sodium bicarbonate supplemented with 0.5 mM sodium pyruvate and 0.05 mM non-essential amino acids. CTX TNA2 cells were maintained in DMEM medium with 4 mM L-glutamine adjusted to contain 1.5 g/L NaHCO_3_ and 4.5 g/L glucose. All media contained 10% FBS and 100 U/mL penicillin. All cells cultures were incubated at 37 °C in a humidified atmosphere of 5% CO_2_. Cells were passaged every 2 days to obtain exponential growth.

### 2.3. Cell Proliferation Assay

Cell viability was determined by MTT assay. Cells (2 × 10^4^ cells/well) were seeded in 24-well plates, incubated for 24 h, then treated with TSAIII at various concentrations (0, 5, 10, 15, 20 μM) for 24 or 48 h. Cells then were incubated in 0.5 mg/mL MTT reagent for 4 h at 37 °C. The cells then were incubated in 1 mL isopropanol at room temperature and the absorbance measured at 570 nm with a Multiskan MS ELISA reader (Labsystems, Helsinki, Finland).

### 2.4. Colony Formation Assay

Cells were seeded into 6-well plates (4 × 10^4^ cells/well). After 24 h, the cells were treated with TSAIII at various concentrations (0, 5, 10, 15, 20 μM) for 7 days. Cells were fixed with methanol for 30 min and then stained with Giemsa solution overnight. The colonies were photographed and counted.

### 2.5. Apoptosis Assay with Annexin V/PI Staining 

The apoptosis assay was performed using the Annexin V-FITC/PI Apoptosis Detection Kit (BD Biosciences, San Jose, CA, USA). GBM8401 and M059K cells were seeded in 6-well plates (4 × 10^4^ cells/well) and treated with TSAIII at various concentrations (0, 5, 10, 15, 20 μM) for 24 h. Cells were collected and fixed and then stained in binding buffer with Annexin V and dead cell reagent for 15 min in the dark. Dead cells were detected using a Muse cell analyzer (EMD Millipore, Billerica, MA, USA).

### 2.6. Mitochondrial Function Assay with JC-1 Staining

GBM8401 and M059K cells were plated on 6 cm dishes (5 × 10^5^ cells/well) and treated with various concentrations of TSAIII (0, 5, 10, and 15 μM) for 24 h. To the cells was added MitoPotential reagent (95 μL) and 7-AAD solution (5 μL), followed by incubation at 37 °C for 20 min and analysis of mitochondrial membrane potential using the Muse cell analyzer (EMD Millipore, Billerica, MA, USA).

### 2.7. Detection of Autophagy with AO Staining 

The autophagy assay was performed as previously described [27]. GBM8401 and M059K cells were seeded into 6-well plates (4 × 10^4^ cells/well) and treated with various concentrations of TSAIII. After 24 h, acridine orange reagent was added to the medium to a final concentration of 1 μg/mL, and the cells were further incubated at 37 °C for 30 min. Cells were then harvested, washed twice with PBS, and analyzed using a FACSCalibur flow cytometer (Becton, Dickinson, and Company, San Jose, CA, USA) with Cell Quest Pro software version 6.0.

### 2.8. siRNA Transfection Assay

Briefly, GBM8401 cells were cultured in 6 cm dishes, incubated to approximately 80% confluence, and then transfected with siRNA-LC3. After 24 h of transfection, cells were treated with or without TSAIII (15 μM) for another 24 h. LC3-II protein expression was analyzed by Western blotting.

### 2.9. Western Blot Analysis

Total protein was extracted from TSAIII-treated glioma cells, and equal amounts of total protein (20 μg) were separated by sodium dodecyl sulfate-polyacrylamide gel electrophoresis (SDS-PAGE) and transferred to PVDF membranes for 70 min. Membranes were blocked with 5% nonfat dry milk buffer, incubated with the primary antibodies, including anti-cleaved-caspase-9 (1:1000), cleaved-caspase-3 (1:1000), anti-cleaved-PARP (1:1000), cytochrome c (1:1000), Mcl-1 (1:1000), p62 (1:1000), LC3-II (1:1000), LAMP (1:1000) and β-actin (1:2000).at 4 °C overnight, and then incubation with secondary antibodies for 2 h at room temperature. Proteins were visualized with ECL reagent (EMD Millipore, Billerica, MA, USA) using the LAS-4000 mini luminescent image analyzer (GE, PA, USA) as described in a previous study [28]. Protein levels are represented as mean ratio values quantified from protein bands of each marker versus β-actin compared to TSAIII-treated cells was performed using ImageJ software.

### 2.10. In Vivo GBM8401 Xenograft-Bearing Mice and GBM Orthotopic Mice Model

In vivo GBM8401 xenograft experiments were implemented under the guidance of the Institutional Animal Care and Use Committee and in accordance with the institutional animal welfare guidelines of Buddhist Dalin Tzu Chi Hospital (IACUC number: 1091101. Data: 2 January 2021). Five-week-old female BALB/c nude mice were purchased from the National Laboratory Animal Center (Tainan, Taiwan). GBM8401 cells (5 × 10^6^ in 100 μL PBS) were subcutaneously injected into the right hind flank of mice. The mice were then divided into three groups (5 mice per each group) according to TSAIII treatment: (1) control (0.1% DMSO diluted in PBS), (2) low-dose TSAIII (5 mg/kg), (3) high-dose TSAIII (10 mg/kg). TSAIII was administered orally three times/week. The tumor volume was measured every 7 days and calculated by the formula (mm^3^) = 0.5236 × L × W^2^. Body weight was measured every 7 days. In vivo GBM8401 orthotopic mice model, the female BALB/c nude mice were anesthesia with Zoletil (i.p. 7.5 mg/100 g) and placed in a stereotactic head frame (Harvard Apparatus, Holliston, MA), GBM8401 (4 × 10^5^/10 μL) was injected into the right striatum of the brain (1 mm forward and a depth of 3 mm). The injected GBM8401 cells times were defined as 0 days. After 1 week, orally with the control group (0.1% DMSO diluted in PBS, *n* = 3), low-dose TSAIII (5 mg/kg, *n* = 3) and high-dose TSAIII (10 mg/kg, *n* = 3) twice a week. At 35 days (xenograft mice) and 21 days (orthotopic mice), the mice were sacrificed under anesthesia, and tumor tissues were weighed and fixed with 10% formalin.

### 2.11. Immunohistochemical Analysis

Formalin-fixed tumor tissues from the TSAIII-treated mice group were embedded in paraffin-embedded tissue 5 µm sections. The slides were heated at 70 °C for 30 min, dewaxing with xylene and alcohol (3 cycles) for 10 min, deparaffinization, and rehydration. Then the slides were treated for antigen retrieval in a citrate buffer (pH 6) for 10 min at 95 °C (DAKO PT Link, Glostrup, Denmark). The primary antibodies were anti-Ki-67 (DAKO, M7240). Tissues Samples were incubated and visualized using Polink-2 HRP Plus Rabbit DAB Detection System (Golden Bridge International, Inc., Mukilteo, WA, USA) for 30 min. The staining areas of tissue sections were observed under a light microscope (Nikon, NY, USA).

### 2.12. Statistical Analysis

Differences in experimental results were analyzed by one-way analysis of variance (ANOVA) followed by the Dunnett post hoc test, and an unpaired 2-tailed Student’s *t*-test was used to determine the significance of differences. Each experiment was repeated three times, and data are presented as the mean ± SE, with *p* < 0.05 or < 0.01 considered statistically significant.

## 3. Results

### 3.1. TSAIII Decreases Viability and Inhibits Proliferation of Human Glioma Cells

**Figure 1:**
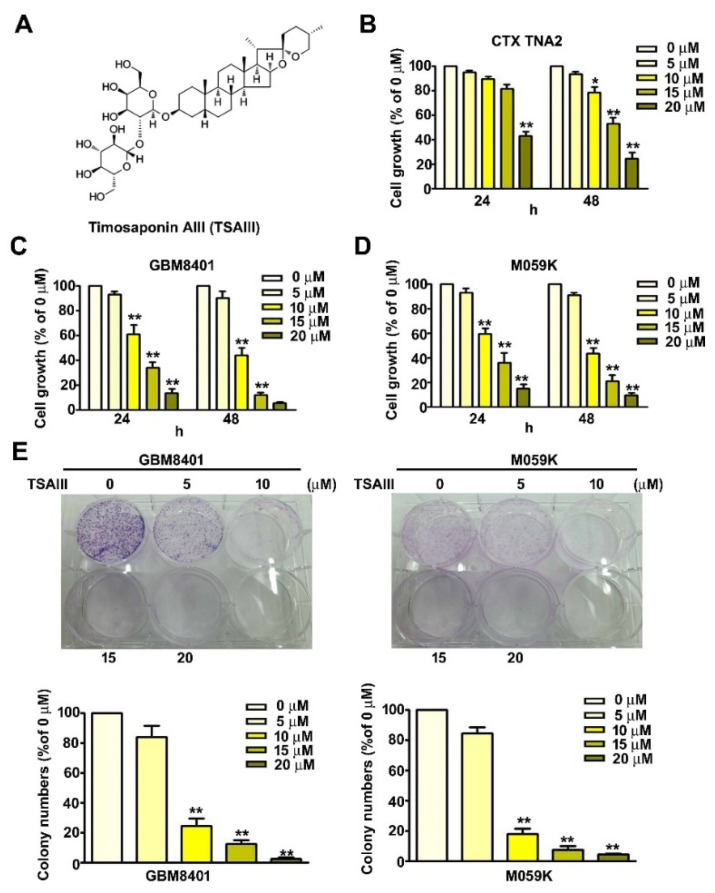
TSAIII decreased viability and inhibited the proliferation of human glioma cells. (**A**) TSAIII structure; (**B**) normal astrocyte (CTX TNA2) cells and (**C**,**D**) human glioma cells (GBM8401 and M059K) were treated with various concentrations of TSAIII for 24 or 48 h. Cell growth was determined by MTT assay. (**E**) Proliferation rates were determined using the colony-forming assay. Each experiment was repeated three times, and data are presented as the mean ± SE. * *p* < 0.05; ** *p* < 0.01 vs. untreated cells.

The structure of timosaponin AIII (TSAIII) is shown in Figure 1A. MTT assay of cells treated with TSAIII for 24 or 48 h showed that TSAIII significantly reduced the viability of GBM8401 (Figure 1C) and M059K cells (Figure 1D) but not CTX TNA2 cells (Figure 1B). The IC50 values of TSAIII on CTX TNA2, GBM8401, and M059K cells were estimated as 18.9, 13.9, and 14.1 μM for 24 h, and 16.2, 9.5, and 8.9 μM for 48 h, respectively. Colony formation assays of human glioma cells (GBM8401 and M059K) treated with TSAIII for 7 days showed that TSAIII markedly suppressed GBM8401 and M059K cell proliferation (Figure 1E, *p* < 0.01 as compared to control). 

### 3.2. TSAIII Induces Apoptosis of GBM8401 and M059K Cells

**Figure 2:**
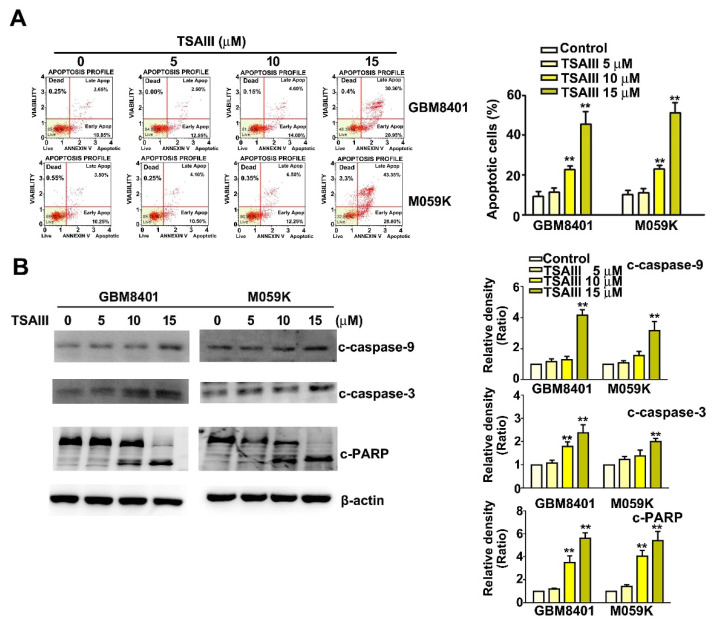
TSAIII induced cell apoptosis and caspase pathways in human glioma cells. (**A**) Human glioma cells (GBM8401 and M059K) were treated with various concentrations of TSAIII (0, 5, 10, 15 μM) for 24 h, harvested, and assayed for apoptosis using Annexin V/ flow cytometry. (**B**) Western blot analysis of c-caspase-3, c-caspase-9, and c-PARP-protein expression in TSAII-treated cells. Quantification of band density was performed using ImageJ. Each experiment was repeated three times, and data are presented as the mean ± SE. ** *p <* 0.01 vs. untreated cells (control).

Flow cytometry of Annexin V-FITC/PI double-stained TSAIII-treated GBM8401 and M059K cells showed that TSAIII induced apoptosis of these cells (Figure 2A). Western blot assay revealed that TSAIII significantly increased the expression of cleaved-caspase-3 (c-caspase-3), cleaved-caspase-9 (c-caspase-9), and cleaved-caspase-PARP (c-PARP) in both glioma cell lines (Figure 2B, *p* < 0.01 as compared to control). These results suggest that TSAIII induced caspase activation of the apoptosis pathway in GBM8401 and M059K cells. 

### 3.3. TSAIII Induces Mitochondrial Dysfunction, Increases Cytochrome C Expression, and Decreases Mcl-1 Expression

**Figure 3:**
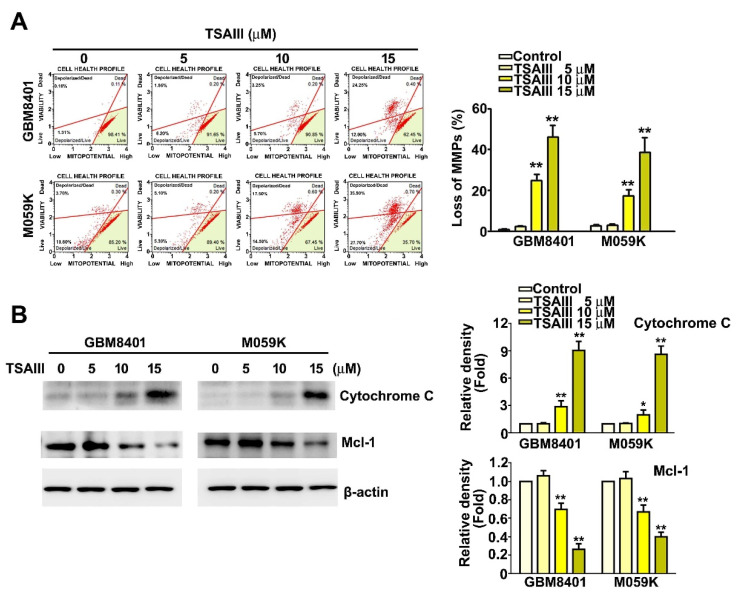
TSAIII induces mitochondrial dysfunction and expression of mitochondria-related proteins in human glioma cells. (**A**) Human glioma cells (GBM8401 and M059K) were treated with TSAIII (0, 5, 10, 15 μM) for 24 h. Harvested cells were assessed for MMPs (mitochondrial membrane potentials) using MitoPotential reagent with flow cytometry. (**B**) Western blot assay of protein expression of Cytochrome C and Mcl-1 in GBM8401 and M059K cells treated with various concentrations of TSAIII (0, 5, 10, 15 μM) for 24 h. Quantification of band density was performed using ImageJ. Each experiment was repeated three times, and data are presented as the mean ± SE. ** *p <* 0.01 vs. untreated cells (control).

To investigate the mechanism underlying TSAIII-induced apoptosis, cell mitochondrial function was assessed after treatment with various concentrations of TSAIII for 24 h. We observed that TSAIII significantly decreased the MMPs (mitochondrial membrane potentials) of apoptotic cells in a dose-dependent manner, indicating that TSAIII caused mitochondrial dysfunction in GBM8401 and M059K cells (Figure 3A, *p* < 0.01 as compared to control). Western blot analysis revealed that TSAIII treatment resulted in significantly up-regulated expression of proapoptotic cytochrome C protein and down-regulation of anti-apoptotic Mcl-1 protein in GBM8401 and M059K cells (Figure 3B, *p* < 0.01 as compared to control). These results suggest that TSAIII induced the mitochondrial apoptosis pathway in GBM8401 and M059K cells.

### 3.4. TSAIII Induces Autophagy in Glioma Cells

**Figure 4:**
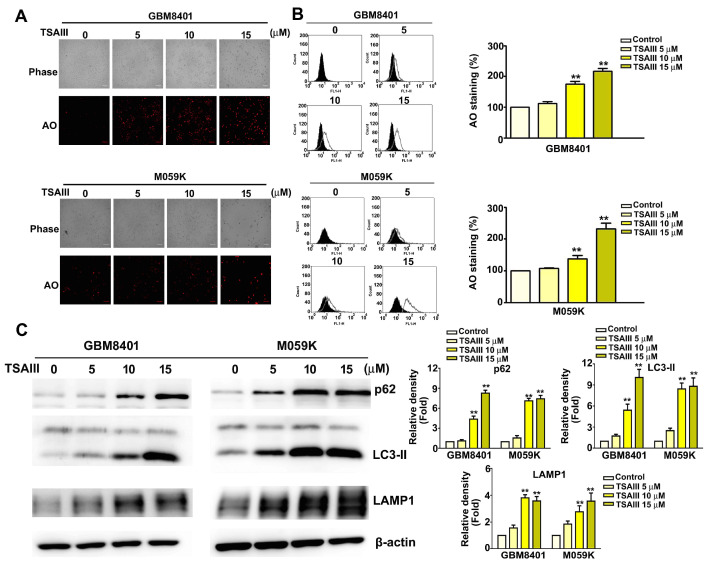
TSAIII induces autophagy and autophagy-related protein expression in human glioma cells. (**A**) Representative fluorescence micrographs of AO staining in GBM8401 and M059K cells treated with various concentrations of TSAIII (0, 5, 10, 15 μM) for 24 h. (**B**) Quantitative results of flow cytometry with AO staining. (**C**) Western blot analysis of protein expression of the autophagy markers p62 and LC3-II and the lysosome marker LAMP1. Quantification of band density was performed using ImageJ. Each experiment was repeated three times, and data are presented as the mean ± SE. ** *p* < 0.01 vs. untreated cells (control).

To investigate the mechanism underlying TSAIII-induced autophagy, GBM8401 and M059K cells were treated with various concentrations of TSAIII for 24 h, stained with acridine orange, and the fluorescence intensity was measured. We observed that the fluorescence intensity increased in a dose-dependent manner over that of control cells (Figure 4A). Quantitative results are presented in Figure 4B. The cytoplasmic form of LC3 (LC3-I) is converted into the autophagosomic form (LC3-II). Western blot analysis shows that treatment of GBM8401 and M509K cells with TSAIII resulted in a dose-dependent increase in protein expression of the autophagy markers p62 and LC3-II and lysosome marker LAMP1 (Figure 4C, left panel, *p* < 0.01 as compared to control). Quantitative results are presented in Figure 4C (right panel, *p* < 0.01 as compared to control). These results suggest that TSAIII induces autophagy in GBM8401 and M059K cells.

### 3.5. Inhibition of Autophagy Increased TSAIII-Induced Apoptosis in GBM8401 and M059K Cells

**Figure 5:**
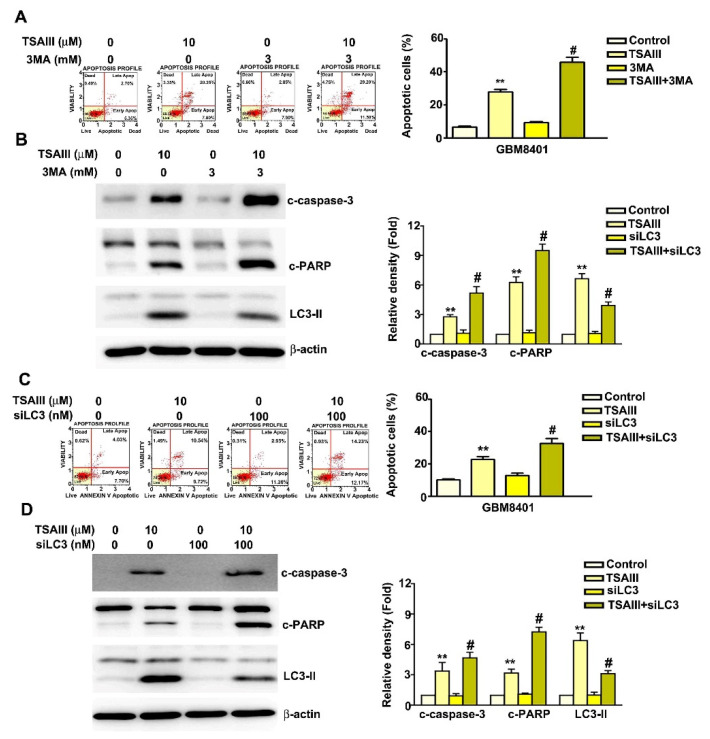
Inhibition of autophagy induced by TSAIII increased apoptosis in human glioma cells. (**A**) Apoptosis assay using Annexin V and flow cytometry of GBM8401 cells treated with TSAIII alone (15 μM), 3MA alone (3 mM), or combined treatment (TSAIII + 3MA) as indicated for 24 h. (**B**) Western blot assay of c-caspase-3, c-PARP, and LC3-II protein expression. (**C**) GBM8401 cells treated with TSAIII alone (15 μM), siLC3 (10 nM) alone, or combined treatment (TSAIII + siLC3) for 24 h, then assayed with Annexin V and flow cytometry. (**D**) Western blot assay of c-caspase-3, c-PARP, and LC3-II protein expression. Quantification of band density was performed using ImageJ. Each experiment was repeated three times, and data are presented as the mean ± SE. ** *p <* 0.01 vs. untreated cells (control). # *p* < 0.05 vs. TSAIII-treated cells.

Annexin V-FITC/PI double-stain assays revealed that cotreatment with TSAIII (15 μM) and the autophagy inhibitor 3MA resulted in a significantly greater number of apoptotic cells than treatment with TSAIII alone (Figure 5A). TSAIII/3MA cotreatment significantly decreased LC3-II expression and increased the expression of cleaved-caspase-3 and cleaved-PARP expression in GBM8401 cells (Figure 5B, *p* < 0.01 as compared to control or TSAIII (10 μM)-treated cells). Silencing of LC3 was used to clarify the mechanism underlying the relationship between autophagy and TSAIII-induced apoptosis. We observed that TSAIII treatment combined with si-LC3 significantly induced apoptosis over that of cells treated with TSAIII (10 μM) alone (Figure 5C, *p* < 0.01 as compared to control or TSAIII (10 μM)-treated cells) and up-regulated the expression of c-caspase-3 and c-PARP (Figure 5D, *p* < 0.01 as compared to control or TSAIII (10 μM)-treated cells). These results suggest that the suppression of autophagy increased TSAIII-induced apoptosis in GBM8401 cells.

### 3.6. TSAIII Inhibits Tumor Growth of GBM8401-Xenograft-Bearing Mice and Orthotropic Glioma Mice Model

**Figure 6:**
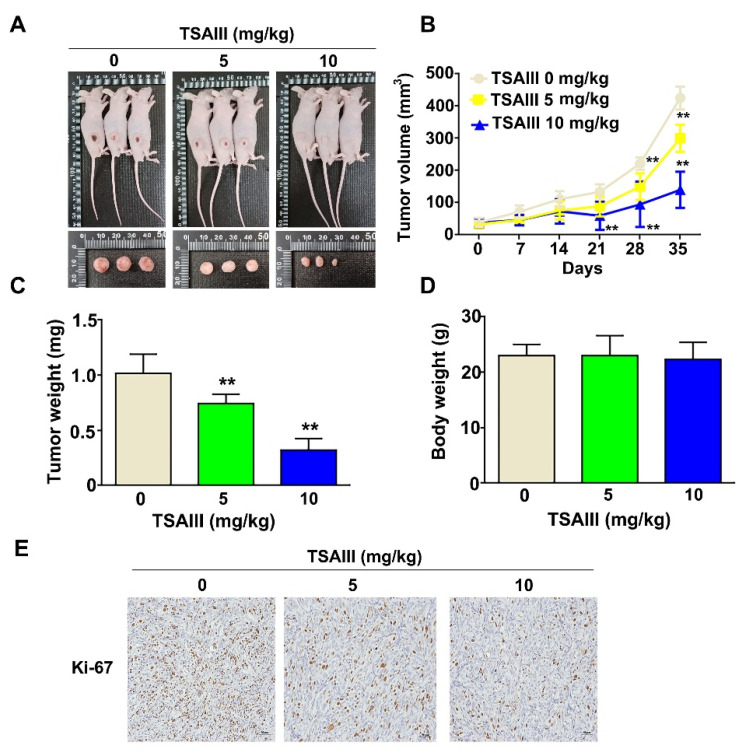
Antitumor effects of TSAIII in GBM8401-xenograft mice. Five-week-old female BALB/c nude mice were treated with normal PBS (control), low-dose TSAIII (5 mg/kg, 3 times/week), or high-dose TSAIII (10 mg/kg, 3 times/week) for 35 days. (**A**) Images of representative tumor tissues from each group; (**B**) tumor volume was measured one time per week until 35 days; (**C**) tumors weight for each group; (**D**) body weight for each group; (**E**) immunohistochemically staining with Ki-67 antibody in paraffin-embedded tumor sections from each group. Data are represented as the mean ± SE. ** *p* < 0.01 vs. control group.

To confirm the in vitro findings in vivo, we subcutaneously inoculated human GBM8401 cells into 5-week-old nude mice as a xenograft-bearing mouse model. Analysis of tumor tissue from GBM8401-xenograft-bearing mice treated with TSAIII (5 or 10 mg/kg) for 35 days revealed that TSAIII treatment significantly reduced tumor size compared to that of untreated mice (Figure 6A). Treatment with TSAIII significantly decreased the tumor growth rate (Figure 6B, *p* < 0.01) and tumor weight (Figure 6C, *p* < 0.01) compared to mice in the control group. Body weight was unaffected by treatment (Figure 6D). Immunohistochemical staining showed that cell proliferation according to Ki-67 expression was lower in TSAIII-treated mice (5, 10 mg/kg) than in the control group (Figure 6E). 

**Figure 7:**
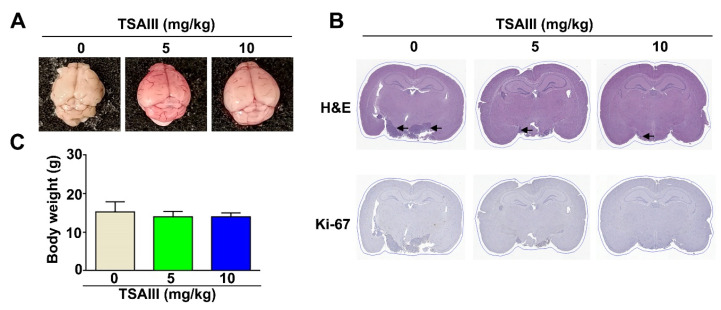
Tumor growth inhibition in orthotopic GBM8401 nude mice by TSAIII. (**A**) Orthotopic glioma tumor formation in the brain of nude mouse. (**B**) H&E staining was determined with histology, and the ki-67 expression was detected with an immunohistochemistry assay in orthotopic glioma tumors. (**C**) Body weight was measured one time per week until 21 days. The position of tumor formation is arrowed.

To further suggest the antitumor role of TSAIII, we established orthotopic GBM8401 nude mice and found that TSAIII significantly inhibited the GBM8401 tumor on the surface of the brain for both doses group (5 and 10 mg/kg), compared with the control group (Figure 7A). Hematoxylin and eosin (H&E) staining of brain tissues suggested effective suppresses in TSAIII-treated with tumor mass, compared with control groups (Figure 7B, upper). However, TSAIII obviously decreased the Ki-67 expression in both doses group (5 and 10 mg/kg) (Figure 7B, down) with no significant change in body weight (Figure 7C). Taken together, these findings demonstrate that TSAIII has the potential to reduce tumor growth in glioma-bearing mice and in orthotopic GBM8401 nude mice.

## 4. Discussion

In this study, we observed the following effects of TSAIII on human glioma cells: (1) decreased viability and proliferation; (2) increased apoptosis; (3) mitochondrial effects, including loss of membrane potential, increased cytochrome C expression, decreased Mcl-1 expression; (4) autophagy induction; (5) increased TSAIII-induced apoptosis in response to autophagy inhibition; and (6) reduced tumor growth in vivo in GBM8401 xenograft mice and in orthotopic mice. These findings show that TSAIII inhibits glioma tumorigenesis by promoting autophagy-related cell death.

Traditional Chinese medicine has been used to cure illnesses and is regarded as a crucial source for the creation of new drugs to combat a variety of cancers [29]. Evidence suggests that the steroidal saponin TSAIII can induce apoptosis and autophagy in a variety of tumor cell lines [30], including HCC [31], acute myeloid leukemia [32], HeLa from human cervical carcinoma [33], breast cancer [30], melanoma [26] and colorectal cancer [34]. Apoptosis progresses through either the intrinsic or extrinsic pathway. The intrinsic pathway results in a loss in mitochondrial membrane potential; increased expression of cyt c, apoptosis-inducing factor, and mitochondrial endonuclease polyG; and activation of caspase-9, caspase-3, and PARP cleavage [35]. In colorectal cancer HCT-15 cells, TSAIII treatment was found to induce apoptosis, as demonstrated by the activation of caspase, induction of cleaved-caspase-3, caspase-8, and caspase-9, and decreased expression of Bcl-xL and Bcl-2 [36]. Another study demonstrated TSAIII-induced apoptosis of human leukemia HL-60 cells via cleaved-caspase-3, caspase-8, caspase-9, and PARP in a dose- and time-dependent manner [32]. In hepatoma HepG2 cells, TSAIII treatment is reported to activate cleaved-caspase-3, caspase-8, and caspase-9, increase the expression of Mcl-1 and Bcl-2, and induce the release of cytochrome C, suggesting that TSAIII induces caspase-dependent and mitochondrial-mediated apoptosis [31]. Our results show that the apoptotic pathway was activated by TSAIII, greatly increasing cytochrome c expression and decreasing Mcl-1 expression. This finding demonstrates that TSAIII inhibits anti-apoptotic protein expression, thereby inducing apoptosis. According to the in vitro results, TSAIII also exhibited a significantly effective inhibition of glioma cell growth in vivo. It has been reported that TSAIII significantly improved scopolamine-induced mice memory impairment [37]. TSAIII up-regulated the acetylcholine contents, inhibited the AChE activity and decreased the expression of TNF-α and IL-1β in scopolamine-treated mice brain models [38], meaning that TSAIII may be across the blood–brain barrier (BBB) in Alzheimer’s disease. Based on our observation, antitumor effect of TSAIII in an orthotopic glioma mice was suggested in vivo. Therefore, TSAIII may be penetrate BBB to the site of orthotopic GBM.

Autophagy plays an important role in maintaining intracellular proteins. In autophagy, aggregated and misfolded proteins and damaged organelles form into autophagosomes, which then fuse with a lysosome. Lysosomes, where cellular waste is eliminated, then promote cell death by recycling defective compartments and cytoplasmic components in double-membrane vesicles, leading to suppression of tumor progression [39]. Recent studies have shown that combining an autophagy inhibitor with commonly used natural compounds, chemotherapy, and targeted drug therapy can inhibit autophagy or induce autophagic cell death activity in various tumor cells [40]. In doing so, inhibition of autophagy can increase the efficacy of antitumor therapy. Previous research has found that TSAIII induces autophagy in breast cancer cells, as evidenced by an increase in LC3-II expression [30]. In the human melanoma cell line A375-S2, TSAIII was found to induce both apoptosis and autophagy through the JNK/ERK signaling pathway [26]. According to a recent study, TSAIII causes HeLa cells to produce cytochrome c and activate caspase-3, both of which indicate an apoptotic state. TSAIII-induced autophagy occurs earlier than apoptosis, as evidenced by the translocation of GFP-LC3 and an increase in LC3 in the absence of cleaved-caspase-3 [33]. We observed here that TSAIII induces autophagic apoptosis in GBM8401 and M059K cells through the up-regulation expression of p62, LC3-II, and LAMP.

Several drug treatments can induce autophagic cell death or both autophagic death and apoptosis [41]. Thus, the exact function of autophagic death appears to be highly dependent on environmental conditions and specific cell types. In lung cancer A549 and H1299 cells, TSAIII mediates autophagy through activation of the AMPK pathway and mediates apoptosis through the MAPK/ERK pathway. In addition, TSAIII treatment has been observed to increase the antitumor activity of 3MA therapy and inhibit autophagy while increasing apoptosis [42]. During tumorigenesis progression, autophagy can play a negative or positive functional role and acts as the “double-edged sword” in tumor cells. Thus, understanding the role of the relationship between autophagy and apoptosis could lead to an effective approach to cancer treatment. Various studies have revealed that inhibition of autophagy by 3-MA rescues MM (multiple myeloma) cells from formosanin C-induced apoptosis [43]. On the contrary, blocking autophagy with CQ (chloroquine) could increase Ginsenoside compound K (CK)-induced neuroblastoma cell apoptosis, indicating the protective role of autophagy in CK-treated neuroblastoma cells in vitro and in vivo [13]. Another report suggested that treated with CQ or specific small interfering RNA (siRNA) targeting Atg5 or Beclin 1 could promote quercetin-induced cell apoptosis against gastric cancer [44]. Consistent with previous reports observed with 3-MA or siRNA-LC3, pre-treatment with TSAIII in GBM8401 cells showed this combination treatment increased the proportion of apoptotic cells, up-regulated the expression of cleaved-caspase-3 and cleaved-PARP, and down-regulated the expression of LC3-II to a greater extent than TSAIII treatment alone. Taken together, these results show that TSAIII-induced autophagy serves a cytoprotective function in glioma cells. It is worth noting that this study only investigated the antitumor inhibitory ability of TSAIII in vivo in glioma-bearing animal models without comparing it to combined therapy with 3-MA (autophagy inhibitor). Therefore, in this study, it was not possible to determine whether the blockage of autophagy induces apoptosis in glioma cells at the in vivo animal level. Further studies using the in vivo intracerebral orthotropic glioma xenograft model to clarify the antitumor effects and molecular mechanisms of TSAIII combined with 3-MA are needed. Our results provide a rationale for further evaluation of TSAIII against glioma by in vivo and clinical studies

## 5. Conclusions

These findings suggest that blockage of autophagy promotes TSAIII-induced apoptosis of glioma cells. With the aim of achieving efficacious treatment for glioma, further research is warranted to determine the antitumor effects of TSAIII/3MA combination treatment in glioma-bearing and in orthotopic glioma mice models.

## Data Availability

The authors will freely release all data supporting the published paper upon direct request to the corresponding author.
